# Reliability of renal point-of-care ultrasound (POCUS) performed by pediatric postgraduates to diagnose hydronephrosis in infants

**DOI:** 10.3389/fped.2024.1361223

**Published:** 2024-04-09

**Authors:** Eun Jung Cheon, Jung Min Yoon

**Affiliations:** ^1^ Department of Pediatrics, Chungbuk National University Hospital, Cheongju, Republic of Korea; ^2^ Department of Pediatrics, Konyang University Hospital, Daejeon, Republic of Korea

**Keywords:** POCUS, medical education, pediatric education, infant—age, hydronephrosis

## Abstract

**Purpose:**

Point-of-care ultrasound (POCUS) has gained prominence in a variety of medical specialties due to advances in ultrasound technology. POCUS has not been fully integrated into pediatric residency training programs despite its widespread use and proven benefits. At our institution, renal POCUS is performed by pediatric residents for the evaluation of hydronephrosis, which is the main pathology for which ultrasound is used in the clinical practice of pediatric nephrology. This study was conducted to evaluate the quality of renal POCUS performed by pediatric residents in infants.

**Methods:**

Four pediatric residents, comprising two first-year and two second-year residents at Konyang University Hospital, participated in the study conducted from May 2021 to May 2022. All participants had completed our Point-of-Care Ultrasound (POCUS) training program. The study focused on infants admitted to the pediatric inpatient unit, identified by attending physicians as requiring renal ultrasound. All infants underwent their initial kidney ultrasound examination. Temporal alignment between renal Point-of-Care Ultrasound (POCUS) performed by pediatric residents and conventional ultrasound (USG) conducted by radiologists was asynchronous. Pediatric residents conducted POCUS sessions during scheduled radiologist appointments throughout the day, occurring either before or after the radiologist's examination. There was no mutual awareness of each other's results. Inter-observer agreement between radiologists and pediatric residents was compared for the presence or absence of hydronephrosis and its grade, which are primary considerations in pediatric renal ultrasound.

**Results:**

Our study found that 53 infants (68.8%) were diagnosed with hydronephrosis using point-of-care ultrasound (POCUS), compared to 48 infants (62.3%) diagnosed with conventional ultrasound (USG). Among the POCUS examinations conducted by pediatric residents, hydronephrosis of SFU grades 1, 2, 3, and 4 were observed in 56.6%, 35.8%, 7.5%, and 0%, respectively. Inter-observer reliability between POCUS and conventional USG showed good agreement, with Cohen's kappa coefficients exceeding 0.8 for sensitivity and 0.6 for grading.

**Conclusions:**

Renal POCUS performed well in diagnosing and grading hydronephrosis in infants when performed by pediatric residents who had completed a two-phase training program.

## Introduction

Point-of-care ultrasound (POCUS) allows clinicians to acquire and interpret images directly at the bedside, empowering them to make real-time, data-driven clinical decisions without relying on a specialist for image acquisition or interpretation (1). This approach is particularly beneficial in critical care and emergency settings. POCUS enables safe procedures and allows for rapid, convenient serial reassessments to enhance diagnosis and monitoring without the need for specialist intervention in image acquisition or interpretation (2). In recent years, it has been widely used in various clinical settings due to enthusiastic reports highlighting its benefits for students, clinicians, and patients alike. Its versatility and effectiveness have made it an indispensable tool, revolutionizing the way medical professionals approach diagnosis and treatment. The advancement of ultrasound technology requires a thorough evaluation of the benefits and risks associated with inadequate training, oversight, and use in imaging-focused medical education programs (3–5).

In pediatrics, POCUS shows great promise. It has an improved acoustic window compared to adult ultrasound and requires less sedation than computed tomography or magnetic resonance imaging. POCUS has also been shown to be useful in pediatric settings, particularly in the emergency department, intensive care unit, and neonatal intensive care unit (6). Despite its potential for expansion into pediatric primary care, POCUS has not yet been fully integrated into pediatric residency training programs (7, 8). This highlights a gap in POCUS education within pediatric residency programs. In one study, the majority of pediatric residents (56%) believed they should receive POCUS training, but only a minority (26%) reported using POCUS in clinical practice. The majority of pediatric residency training programs do not provide POCUS training to their residents, despite the value and importance of POCUS training (9, 10).

Recently, we introduced renal POCUS training into our pediatric residency program. The goal of this training program was to provide pediatric residents with the skills necessary to independently perform renal POCUS on live patients. Beginning with the completion of two video lectures on basic renal anatomy and renal ultrasound techniques, residents followed a structured process. The video lectures were selected and provided by the pediatrics residency program. Only residents who completed the first step were eligible to participate in the next step. In the second phase, residents were required to perform hands-on renal POCUS for at least 1 month while supervised by an attending physician. This step required a minimum of 10 renal POCUS per resident and required a “pass” approval from the attending physician. Pediatric residents who performed well in steps 1 and 2 could subsequently perform renal POCUS on live patients independently. The supervising physicians were certified as clinical sonographers by the Korean Society of Clinical Ultrasound. Our study aimed to assess the reliability of POCUS performance of residents who completed our renal POCUS training program designed to train pediatric residents.

## Materials and methods

### PICO (problem/population, intervention, comparison, outcome)

Our study was conducted within the following framework (Box 1).

BOX 1. **Framework for pediatric renal point-of-care ultrasound (POCUS) in diagnosing hydronephrosis in infants.**

**Table:** 

Element	Description
Population/problem	Hospitalized infants requiring renal ultrasound
Intervention	POCUS performed by a pediatric resident
Comparison	Conventional renal USG performed by a radiologist
Outcome	Performance of renal POCUS performed by pediatric residents is not inferior to conventional renal ultrasonography performed by radiologists

POCUS, point-of-care ultrasound; USG, ultrasonography.

From May 2021 to May 2022, a total of 304 children underwent renal point-of-care ultrasound (POCUS) conducted by pediatric residents. Out of these, 77 infants (53 boys, 24 girls; mean age 6.5 months, range 1–9.3 months) were included in the study. Four pediatric residents, two from each year of the program at Konyang University Hospital, participated in the study participated in the study after completing our two-stage renal POCUS training and providing informed consent participated in the study after completing our two-stage renal POCUS training and providing consent. Included cases involved infants who were deemed by the attending physician to require a renal ultrasound without a previous renal ultrasound. Excluded cases included infants who were unable to complete two consecutive ultrasound scans due to restlessness or who were suspected of having severe renal abnormalities other than hydronephrosis on the initial pediatric POCUS scan.

### Renal POCUS and conventional ultrasonography examination

Subjects were infants admitted to the pediatric inpatient unit who required a renal ultrasound as determined by the attending physician. All infants were undergoing their initial renal ultrasound. The point-of-care ultrasound (POCUS) by the pediatric resident and the conventional ultrasound (USG) by the radiologist were performed independently: if there was an appointment scheduled with the radiologist, the pediatrician would perform the renal POCUS during the day's routine. It could be done before or after the radiologist had performed the test, but they were not aware of the results of the other test.

Ultrasound examinations were performed using linear transducers (L12-5 50 mm) of the iU22 ultrasound system (Philips Ultrasound. Inc. USA). These are known for their high frequency and suitability for imaging small parts and superficial structures. They have a high frequency and are useful for small parts and shallow structures close to the body surface. They produce a rectangular image with a frequency of 5–12 MHz. They examined the shape and length of both kidneys and confirmed the presence and grade of hydronephrosis, the main concern in pediatric renal ultrasound. Pediatric residents performed the examination in the prone position, and radiologists used the supine position.

### Inter-examiner agreement between radiologists and pediatric residents

Inter-examiner agreement between radiologists and pediatric residents regarding the presence and severity of hydronephrosis, a significant complication in pediatric renal ultrasound, was evaluated. Hydronephrosis was graded according to the Society of Fetal Urology (SFU) classification system (9). Intra- and inter-rater reliability could not be determined individually for each resident due to the limited feasibility of infants undergoing repeat ultrasound. Additionally, individual resident performance was not separately compared in this study, as the primary objective was to assess whether the POCUS skills of residents who completed our training program were comparable to those of radiologists.

### Statistical methods

We employed IBM SPSS 26.0 (IBM Co., Armonk, NY, USA) for statistical analysis, encompassing analytical and descriptive statistics along with crosstabulations. Inter-observer reliability, which assesses the agreement between two raters in assigning categorical variables was evaluated using Cohen's kappa coefficient (κ), a statistic commonly utilized to gauge both inter-rater and intra-rater reliability of qualitative assessments (11).

## Results

### Study population

A total of 77 infants, including 53 boys (68%) and 24 girls (32%), were enrolled at an average age of 6.5 months (range: 1 day to 9.3 months). Indications for renal ultrasound included febrile urinary tract infection (24.7%, *N* = 19), oligohydramnios from birth history (6.5%, *N* = 5), suspected renal anomalies detected during antenatal screening (4%, *N* = 3), and electrolyte abnormalities along with minor urinalysis abnormalities identified during admission screening examinations (64.9%, *N* = 50). A kidney ultrasound is not a routine test for children with mild electrolyte or urine abnormalities, but participation in this study will allow children to have a free ultrasound, allowing parents to have a kidney ultrasound if they are concerned about the test results. Exclusions comprised five patients with consecutive failed tests and two patients with suspected severe renal abnormalities. A Formal ultrasonography was requested when renal parenchymal changes other than simple hydronephrosis were detected. This led to the diagnosis of unilateral multi-cystic dysplastic kidney in one patient and horseshoe nephropathy in another.

### Assessment of hydronephrosis

The renal anatomy of the patients was independently evaluated by a radiologist and a pediatric resident to determine the presence of hydronephrosis. Hydronephrosis was graded according to the Society of Fetal Urology (SFU) grading system. Point-of-care ultrasound (POCUS) identified hydronephrosis in 53 infants (68.8%), while conventional ultrasound (USG) diagnosed hydronephrosis in 48 infants (62.3%). Among the POCUS examinations performed by pediatric residents, hydronephrosis of SFU grades 1, 2, 3, and 4 were observed in 56.6% (*N* = 30), 35.8% (*N* = 19), 7.5% (*N* = 4), and 0%, respectively (Table 1). The distribution of SFU grades for these infants was as follows: 58.3% (*n* = 28) for grade 1, 37.5% (*n* = 18) for grade 2, 4.1% (*n* = 2) for grade 3, and 0% for grade 4.

**Table 1:** Diagnosing hydronephrosis using conventional USG by a radiologist and POCUS by a pediatric resident.

	Conventional USG	POCUS
No hydronephrosis	24 (31.2%)	29 (37.7%)
Hydronephrosis		
SFU grade 1	30 (56.6%)	28 (58.3%)
SFU grade 2	19 (35.8%)	18 (37.5%)
SFU grade 3	4 (7.5%)	2 (4.1%)
SFU grade 4	0 (0%)	0 (0%)
Total	53 (68.8%)	48 (62.3%)

POCUS, point-of-care ultrasound; SFU, society of fetal urology; USG, ultrasonography.

### Inter-observer concordance between POCUS and conventional USG

**Figure 1:**
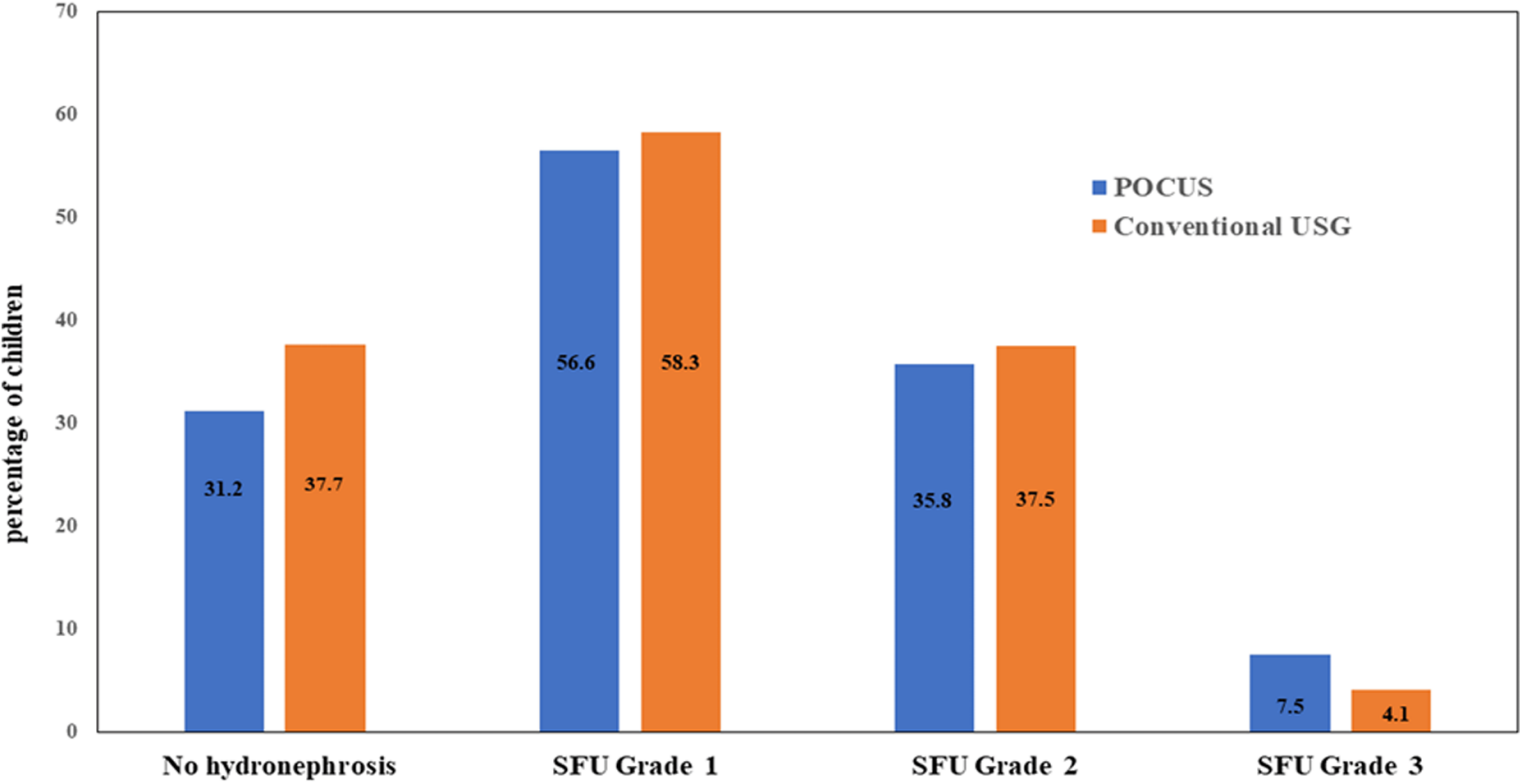
Agreement of POCUS and conventional USG in the examination for the hydronephrosis of infant. POCUS, point of care ultrasound; USG, ultrasonography; SFU, society of fetal urology.

**Figure 2:**
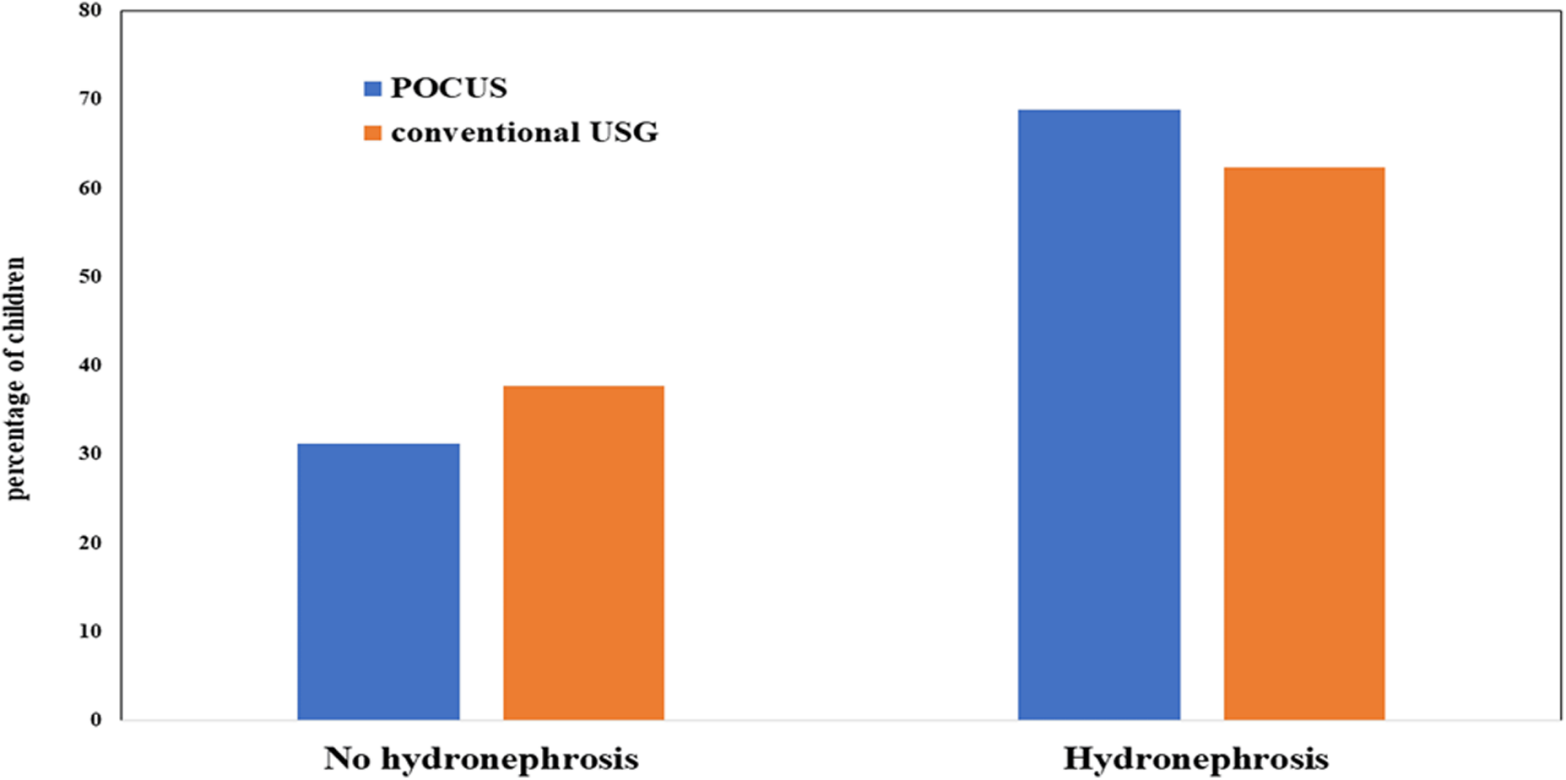
Agreement of POCUS and conventional USG in the examination for the hydronephrosis of infant. POCUS, point of care ultrasound; USG, ultrasonography.

The cross-over analysis affirmed the inter-observer concordance between POCUS and conventional USG, demonstrating a Cohen's kappa coefficient exceeding 0.8 for the initial evaluation, signifying almost perfect concurrence, and a kappa value surpassing 0.6 for the SFU assessment, denoting substantial concordance (*p* < 0.001) (Figures 1, 2). In the diagnosis of hydronephrosis by a radiologist, the gold standard is considered to be the gold standard, POCUS was performed by a pediatric resident who completed the POCUS training program. The sensitivity was 98%, specificity was 79%, positive predictive value was 89%, negative predictive value was 96%, and accuracy was 91%.

## Discussion

While there is a growing demand for integrating Point-of-Care Ultrasound (POCUS) training into pediatric residency programs (12, 13), some pediatricians remain hesitant to independently utilize ultrasound due to their limited experience, often deferring to radiologists. This demand underscores residents' apprehension in conducting POCUS examinations, which stems from insufficient training.

Despite the American Academy of Pediatrics' efforts to establish consensus and guidelines for ultrasound use in pediatric emergency medicine over the last decade, there remains a dearth of standardized curricula tailored specifically for pediatric residents. Although the Korean Society of Pediatric Emergency Medicine has introduced voluntary training programs in pediatric emergency ultrasound, the absence of dedicated curricula for pediatric resident training in POCUS persists, with existing programs still in their nascent stages of development (14). However, there is a lack of curricula dedicated to training pediatric residents in POCUS, and those available are still in their early stages of development (9, 15, 16). Over recent years, we have implemented point-of-care ultrasound (POCUS) training for pediatric residents utilizing available educational resources. Hence, the objective of this study was to assess the effectiveness of our renal POCUS training program by evaluating the competency of pediatric residents in conducting renal ultrasound examinations. The assessment of the presence of hydronephrosis showed substantial agreement, with a kappa coefficient exceeding 0.8, while the assessment of hydronephrosis grading revealed moderate agreement, with a kappa exceeding 0.6. These findings affirm the efficacy of our renal POCUS training program and establish the reliability of renal POCUS conducted by pediatric residents.

Our training program is simple and practical. As part of their training, pediatric residents first familiarize themselves with kidney anatomy by reviewing reconstructed ultrasound images, thereby reinforcing their existing knowledge. Subsequently, they acquire proficiency in ultrasound techniques through online educational modules. The quality of the educational content is carefully selected and delivered by the POCUS educational program, and the selection is determined by the attending physician. For individuals who have successfully completed a traditional medical school program, this step should pose no significant challenge to accomplish. After completing this stage, the resident will conduct a minimum of 10 hands-on renal POCUS examinations within a month, during which the attending physician will assess the performance and imaging outcomes, providing approval to proceed to the subsequent phase. This POCUS program works best in pediatrics when there is an experienced POCUS attending and fewer trainees. The advantage is that the attending knows more about each trainee's training situation and can provide individualized guidance throughout the training process.

In this study, radiologists and pediatricians used different patient positions during renal ultrasound scans. Pediatric residents conducted the examination with the patient in the prone position, while radiologists utilized the supine position. While both positions are deemed appropriate for renal ultrasound, our POCUS training program opted for the prone or lateral decubitus position to enhance examination comfort during its development. Traditionally, radiologists perform renal ultrasound on patients in the prone position, which can lead to interference from gastric air and bowel distention, particularly when examining the left kidney. The kidneys are situated retroperitoneally, making the prone position advantageous for easier access and decreased susceptibility to bowel obstruction. However, patient discomfort in the prone position may limit the examination, in which case transitioning to the lateral decubitus position is recommended.

To the best of our knowledge, there have been no studies of renal POCUS in children. The overall conclusion is that POCUS and USG performed by pediatricians work well together in diagnosis. Our findings might be misconstrued as “overdiagnosis” with POCUS in comparison to conventional USG, particularly if radiologist-performed USG is regarded as the standard. However, a study conducted by Sameer A et al. (17) in a sizable adult cohort compared the accuracy of physician POCUS diagnosis of hydronephrosis with radiologist-performed USG. The study revealed that physician POCUS exhibited relatively low sensitivity, with higher sensitivity only in cases of severe hydronephrosis. Several other researchers have documented analogous results (18–20). As the initial investigation examining the quality of POCUS diagnosis of hydronephrosis in children, presenting pediatric examples proves challenging. However, drawing from previous adult studies, a notable concern in the clinical realm regarding POCUS diagnosis of hydronephrosis by internists is the occurrence of false negatives. Therefore, we do not believe that the relatively high rate diagnosed by pediatricians in our study is concerning. Hence, we advocate for advancing to the subsequent diagnostic phase of referring to a radiologist when POCUS indicates more intricate conditions like renal parenchymal disease or unilateral renal agenesis.

There are studies similar to ours in terms of curriculum design. For instance, Goods et al. (21) proposed a 1-month in-service POCUS training program for pediatric residents during their PICU rotation. The program comprises several components: (1) pre- and post-training knowledge assessments, (2) online self-study modules covering ultrasound fundamentals as well as lung and cardiac ultrasound, (3) two consecutive 1-h hands-on sessions with patients exhibiting normal anatomy admitted during the initial 2 weeks, and (4) two consecutive 1-h POCUS rounds with patients admitted during the initial 2 weeks. This curriculum is suitable for institutions equipped with adequate patient volume, resources, and expertise. Although previous studies have been single-center experimental studies with limited numbers of participants, they have shown that POCUS training correlates with improvements in self-reported POCUS confidence and comfort.

Simulation-based learning programs are integral to medical education, including ultrasound training (22, 23), fostering confidence and proficiency among students and patients. Presently, emphasis is placed on simulation-based ultrasound training, particularly for interventional procedures like gynecologic ultrasound, echocardiography, and vascular access. Despite the growing utilization of point-of-care ultrasound (POCUS), many hospitals lack simulators for training pediatric residents, compounded by limited experience among pediatric faculties. While hospitals acknowledge the necessity of POCUS training, practical constraints often impede its integration.

A limitation of our study is the inclusion of infants under 1 year of age as subjects. The inclusion of infants is justified by the particular challenge posed by their limited ability to cooperate during ultrasound examinations. Mastery of ultrasound techniques in this population can enhance proficiency in the performance of ultrasound examinations in various age groups. Furthermore, the clinical significance of targeting this age group is highlighted by the prevalence of febrile urinary tract infections among patients undergoing their initial renal ultrasound examination, a condition frequently observed in children under the age of 1 (24).

Our POCUS training model has the advantage of being easily adaptable to most hospitals. An essential consideration is the requirement for a preceptor within the pediatric department to serve as the POCUS supervisor, ensuring the applicability of our training model in a pediatric residency program. Michael et al. emphasized the significant challenge posed by this prerequisite, underscoring the widespread lack of familiarity among teaching hospital preceptors with POCUS. According to the study, a lack of adequately trained faculty or instructors is the primary barrier to implementing a point-of-care ultrasound curriculum (70.4%), lack of guidelines or standards endorsed by governing societies (44.4%), and limited access to essential technology (33.3%) (25, 26).

## Conclusion

As an educational resource available in the current situation, we have incorporated Renal POCUS into the training of pediatric residents, and it shows good performance results in diagnosing hydronephrosis in infants, which is the main reason for performing renal ultrasound in pediatrics. We believe that for many hospitals that are unable to implement POCUS in pediatric residency training, our experience can be a positive motivation.

## Data Availability

The raw data supporting the conclusions of this article will be made available by the authors, without undue reservation.
